# The migraine epidemic among medical students: a call for action

**DOI:** 10.1097/MS9.0000000000002241

**Published:** 2024-06-10

**Authors:** Muhammad Saeed, Muhammad Usman Farooq, Mohammad Najaf Ali Abbas, Faheemullah Khan, Djabo Eric Adrien Tangmi, Gaurav Mittal

**Affiliations:** a D. G. Khan Medical College, Dera Ghazi Khan, Pakistan; b Section of Cardiovascular Imaging, Diagnostic Institute, Cleveland Clinic, OH, USA; c Department of Medicine, Université Technologique Bel Campus, Kinshasa, Democratic Republic of Congo; d Department of Research, Mahatma Gandhi Institute of Medical Sciences, Wardha, Maharashtra, India

**Keywords:** migraine and headaches, migraine and young medical students, migraine in medical schools, migraine in medical students, migraine prevalence among young students

## Abstract

Migraine is characterized by recurrent headaches of moderate-to-severe intensity and poses a significant challenge for medical students. This is a narrative literature review using PubMed and Scopus databases. This study examines how common migraine is in this group and suggests working together to address how it affects students’ well-being and chances of succeeding as a medical professional in the future. Early diagnosis by licensed medical specialists is essential for effective management of migraine. To address this, the authors propose a multifaceted strategy. By including direct education on migraines in medical school curricula, future doctors will be better prepared to treat patients with comparable problems and manage their own migraines. Students with migraines can also benefit greatly from creating a supportive learning environment through staff training, accommodating academic policies, and providing easily available healthcare resources. In addition, this technology may be helpful. Apps for relaxation and migraine tracking can help students better manage their condition. Long-term success requires cooperation among all parties. By promoting cooperation among medical schools, student associations, healthcare practitioners, and governmental organizations, the authors can raise public awareness of migraine, make pertinent resources easier to access, and create evidence-based solutions specially designed to meet the needs of medical students who experience migraine. In the end, putting student well-being first and working together to put these solutions into practice can enable aspiring doctors to succeed at both personal and professional levels.

## Introduction

Highlights

- High prevalence of migraine in medical students.
- Multifaceted approach to address the impact of migraine.
- Collaborative effort for long-term solutions.
- Focus on student well-being.

Migraine is a debilitating neurological condition characterized by recurrent headaches. The International Headache Society (IHS) states that migraine attacks usually last four to seventy-two hours and are frequently accompanied by other symptoms such as light and sound sensitivity, nausea, and vomiting^1^. Beyond just the physical discomfort, migraineurs’ lives are profoundly affected. According to previous studies, those who suffer from migraines have a significantly lower health-related quality of life than people who have long-term diseases, such as epilepsy or heart failure^2^. Every year, millions of workdays are missed due to migraines, which results in significant financial losses. According to estimates from the Migraine Research Foundation, decreased productivity costs the American economy more than $36 billion a year. Millions of people are affected by this common ailment worldwide. According to estimates from the WHO, one in seven people suffer from migraine globally. It is estimated that more than 39 million Americans suffer from it^3^.

Compared with the general population, medical students have a remarkably high prevalence of migraine. This is a serious obstacle to academic progress and the general well-being of students. A 2019 study conducted among Chinese medical students revealed a startling 42.3% prevalence of migraine, which is far higher than that in the country as a whole. This issue is not specific to any particular area. A different study published in Headache: The Journal of the American Headache Society in 2017 found that 38.2% of medical students had migraines^4,5^.

The purpose of this review is to shed light on the alarmingly high incidence of migraines among medical students. We will investigate the epidemiology of migraine in this demographic, pinpoint relevant factors, and assess the effects on student well-being by reviewing the recent literature. It will also review current migraine care techniques, point out difficulties, and suggest best practices for helping medical students.

## Methodology

We did a narrative literature review using PubMed, Cochrane and Scopus databases in February-March 2024 for keywords; “Migraine in medical students,” “Migraine in medical schools,” “Migraine and young medical students,” “Migraine prevalence among young students,” and “Migraine and headaches.” We reviewed cross-sectional studies, clinical and controlled trials, pharmacological studies, and articles. The search included articles published in English. Most publications from the last 10 years were selected, but commonly cited and referenced publications were not excluded. The reference lists of relevant and recent primary articles, book chapters, reviews, and alternatives were also reviewed to identify studies that may have been missed.

## The burden of migraine on medical students

For medical students, migraine attacks present a major obstacle that affects their personal and academic lives in different ways. There is a clear link between migraine and the inability to focus during episodes. According to a 2013 study published in BMC Neurology, more than 68% of medical students with migraine reported having trouble concentrating, which can seriously impair their ability to study, finish homework, and perform well on tests^6^. Attendance at lectures, tutorials, and other important learning activities may be disrupted by migraine attacks. This may result in lost knowledge, trouble keeping up with the demanding curriculum, and eventually poorer academic achievement^7^. Participation in clinical rotations, which are crucial for developing practical skills and gaining professional experience, can be severely hampered by migraine attacks. During an assault, students could find it difficult to attend clinical rounds, help with patient care, or finish prescribed assignments^8^. Students who miss rotations due to migraines may experience elevated levels of stress and worry. A vicious cycle may be created by the drive to catch up and the dread of more attacks^9^.

Medical students’ mental health may be affected by the chronic nature of migraines and their fear of attack. Research indicates that medical students who suffer from migraines are more likely to experience anxiety and sadness than those who do not^2,10^. Burnout and a decrease in motivation might result from the ongoing battle to balance it with the rigorous workloads associated with academics and clinicians. Academic achievement and general well-being may be further affected by this. For those who are vulnerable, the stressful atmosphere in medical schools produces an ideal storm for the development of migraines. Numerous elements are likely to come into play^11^.

Owing to intense competition, long hours, and heavy workloads, medical schools are known to be extremely stressful. For susceptible students, this ongoing stress can result in migraine headaches^2^. In medical students, subjective stress and migraine frequency were found to be significantly correlated in a study that was published in the Journal of Clinical Psychiatry in 2000^12^. According to reports, medical students who suffer from migraines tend to experience higher levels of stress than those who do not^13^. Due to the demands of their clinical rotations and coursework, medical students frequently forgo sleep. It is generally known that irregular sleep habits can cause migraines^14^. Compared to medical students without migraines, people with migraines sleep considerably less each night^8^. Additionally, there is a correlation between more screen time and frequent headaches^15^. It can get worse in medical students due to irregular eating regimens, dehydration, and lack of physical activity because of hectic schedules^16^. There may be a link between migraine risk and unhealthful eating habits, according to research^17^.

For medical students, untreated or improperly controlled migraines can have serious repercussions that extend beyond the acute agony of attacks and affect their overall health and well-being. Medication overuse headaches (MOH) can result from excessive reliance on pain medication to treat recurrent bouts. As a result, there is a vicious cycle in which medicine itself starts to cause more headaches. According to reports, medical students who suffer from migraines are more likely than those who do not abuse medications^2^. Medical students may acquire anxiety and sadness as a result of chronic pain and the disturbance it causes^12^. Medical students’ general quality of life is greatly impacted by uncontrollably occurring migraines, which make it difficult for them to fully engage in both personal and academic activities^2,18^.

## Migraine management in medical schools

Unfortunately, there is a paucity of thorough studies that concentrate on the support networks that medical schools provide for migraineurs. However, certain tools and support systems exist based on best practices in student health services and the available resources. Most medical schools probably have student health services that are capable of diagnosing migraines and offering general medical evaluations. It is also possible to refer patients to neurologists or headache specialists for additional assessment and treatment planning. Appropriate drugs for treating and preventing migraines are readily available. Assistance in coping with the stress, worry, and sadness that frequently accompany migraines averts additional health issues^12^.

Students with migraines may benefit from wellness programs offered by several medical schools. Resources and workshops emphasizing mindfulness, time management, and relaxation techniques are beneficial means of lowering migraine triggers. Programs that encourage sound sleeping practices, consistent exercise, and a balanced diet may have an impact on migraine prevention. Information regarding the prevalence of flexible learning settings, particularly for students with migraines, is scarce. On the other hand, certain medical schools may grant extra time for exams when migraines have been shown to affect student performance. They can recover academically if they have access to the recordings of lectures or presentations they missed. Medical schools should encourage students experiencing migraines to speak up for themselves and seek appropriate services for support. Educating faculty and staff about the impact and frequency of migraines can help create a more encouraging workplace^16^.

There are evidence-based gaps and limitations in current migraine management strategies, although certain medical schools may provide some basic tools. According to one study, medical students frequently do not know enough about migraines, including how to properly diagnose them, what treatments are available, and how to prevent them^8^. This ignorance may result in inadequate care, a delayed diagnosis, and the use of potentially dangerous coping techniques. Students may be reluctant to disclose migraines due to the fast-paced and rigorous environment of medical school, fearing they would be viewed as weak or untrustworthy^11^. This quiet can make it more difficult to get the right help and exacerbate feelings of loneliness. Faculty who have had insufficient training or have not had much exposure to migraine education may not be able to identify the difficulties faced by students with migraines. This may make it more difficult to adopt flexible learning arrangements or make the necessary concessions. The primary goal of currently available resources may be to treat the acute pain associated with migraine attacks. A more all-encompassing strategy should include prioritizing stress-reduction methods, preventative measures, and alterations to a healthy lifestyle^2^.

A combination of medications for acute attacks and preventive measures can effectively manage migraines. It is known that long-term stress can provoke migraine. Studies indicate that practices such as progressive muscular relaxation, yoga, and mindfulness meditation can effectively decrease its occurrence^19^. According to reports, practicing mindfulness meditation reduced the frequency and intensity of migraines^20^. Its frequency can also be greatly impacted by sticking to regular sleep schedules, getting regular exercise, and eating a healthy diet. Migraine prevention can be achieved by identifying and eliminating personal food triggers such as aged cheese, processed meats, and alcohol. It can be avoided with several medications such as beta-blockers, anti-epileptic medications, and some antidepressants. Based on a patient’s unique needs and medical history, a physician can assist in selecting the best course of action^21,22^. Healthcare practitioners can use the evidence-based guidelines for migraine prevention published by the American Academy of Neurology (AAN) to inform their treatment decisions^22^. Acute migraine attack pain and other symptoms can be controlled with the use of medications such as triptans and painkillers. Information about different acute migraine drugs and when to take them is available from the American Migraine Foundation^23^. It can also be managed by keeping a headache journal to record possible triggers such as stress, particular meals, hormone shifts, or weather patterns^24^. Massage therapy, acupuncture, and biofeedback therapy can all be relieving for certain people. Research on the efficacy of these strategies is still being conducted^25^. People with migraines can learn coping techniques for handling the tension, anxiety, and discomfort of attacks with the aid of cognitive behavioral therapy (CBT)^26^.

For medical students to manage their migraines effectively and enjoy a higher quality of life, early diagnosis and treatment by healthcare providers are essential. Choosing the best preventative medicine and acute treatment plan is made possible by correct diagnosis. Postponements may result in unnecessary agony and loss of chance for efficient handling. If left untreated, migraine can progress to chronic migraine, which is defined as headaches that occur at least 15 days per month. This can seriously impair the students’ capacity to learn. Migraine may occasionally be a sign of underlying medical issues. Early detection can guarantee that these illnesses are treated appropriately^2^. Medical students may benefit from using technology to control their migraines. Students can keep track of their migraines, pinpoint possible causes, and assess the efficacy of various treatment options using migraine-tracking apps. Talking about this information with medical professionals would be beneficial. Apps for relaxation and meditation that provide guided breathing exercises, mindfulness training, and relaxation methods can be useful aids in reducing stress, which is known to trigger migraine. Technology is a useful tool, but it should not replace consulting a medical practitioner^19^.

## Recommendations for medical schools

Medical schools can implement a multifaceted strategy to address the high incidence of migraine among students. It can be beneficial to attend workshops on migraine management conducted by medical professionals or by seasoned migraine patients. A summary of the various treatment options (acute medications, preventive medications), effective stress management techniques (mindfulness, relaxation exercises), the significance of sleep hygiene for migraine prevention, and the identification and avoidance of migraine triggers (diet, sleep, stress) can all be covered in these sessions^19^. Presentations, online tools, and informational booklets on migraines can increase knowledge and provide students with the tools they need to successfully manage their illness. Teaching professors and staff about migraines can help lessen stigma and encourage students to seek care without fear of judgment. This can also promote empathy and understanding. Students who suffer from migraines may find it helpful to have flexible learning choices, such as access to lecture recordings, assignment extensions, or acceptable absences with supporting proofs. Collaboration with the Student Health Center should be promoted to guarantee that medical specialists qualified to diagnose and treat migraines are available. It can be helpful to have access to the right acute and preventive drugs as well as alternatives for referrals to neurologists or headache specialists as necessary^27^. It should be promoted for students to use migraine-tracking applications to track the efficacy of their treatments and uncover personal triggers. It may be possible to investigate the viability of providing telemedicine consultations with medical specialists specializing in migraine treatment^28^.

Medical schools can help students with migraines succeed academically and maintain their well-being by implementing these strategies. The academic performance and general well-being of medical students can be considerably affected by migraines. Nonetheless, medical schools can overcome these obstacles by incorporating education about migraines, discussing both pharmaceutical and non-pharmacological effective treatment options, and stressing the value of early diagnosis and specialist referral when necessary^8^.

## Collaboration and future directions

Medical students with migraine face a challenging situation that calls for multimodal solutions. Collaboration among stakeholders is essential for efficient management. Medical schools must create a welcoming environment for their students. They can provide flexible learning alternatives, teach staff about the requirements of students, and incorporate migraine education into the curriculum^8^. Peer support, informational resources, and advocacy for student needs can be obtained from student organizations that concentrate on wellness or particular conditions such as migraines. For complex illnesses, qualified medical professionals in student health centers and outside collaborations can offer diagnoses, treatment strategies, and referrals^11^. Governmental organizations that work in higher education or public health may provide funds for studies on migraine in medical students or for activities that enhance student well-being. Medical schools can combine their educational programs with access to healthcare experts, prospective government research grants, and peer support from student organizations. By lowering stigma and encouraging early help-seeking behavior, a collaborative strategy can increase awareness of migraine among students, teachers, staff, and the general public. For children suffering from migraines, this can facilitate the creation and distribution of educational materials, support systems, and necessary healthcare services^2^.

Although much research is being conducted on migraines in general, not much has been done in-depth on medical students in particular. A few possible topics require further research. Research may examine the precise relationship between migraine frequency or intensity and the rigorous academic environment of medical schools^8^. Studies should look into the connection between migraine and sleep habits among medical students, given the particular difficulties they encounter. It is possible to examine the frequency of co-occurring mental health issues, such as anxiety or depression, in medical students who have migraines and how they could affect one another^12^. Studies could assess how well-mindfulness-based stress-reduction programs or meditation applications work to improve coping strategies and lessen migraine frequency in medical students. It is possible to evaluate the viability and efficacy of providing medical students telehealth consultations with migraine specialists. Using efficient migraine treatment in medical students can have a major positive long-term impact on the health of students as well as the next generation of doctors^20^.

A student’s academic life can be less disrupted by migraines when they employ effective management measures, which can enhance both academic achievement and general well-being^2^. It can lessen the frequency and intensity of migraines, enabling adolescents to engage more fully in social and academic activities and, eventually, enhance their quality of life. Better self-care and well-being can be encouraged among students by lowering the stigma associated with migraines and encouraging them to seek help without fear of being judged^11^. Future doctors can be equipped with the information and abilities to manage their own migraines, which will enable them to apply therapeutic and preventive techniques in their own lives and encourage good behaviors throughout their careers^23^. A person’s personal migraine experience can help them empathize with and understand other patients going through similar difficulties, which can result in more effective and compassionate patient treatment. Proficiently handling long-term ailments, such as migraines, can enhance a doctor’s resilience and self-care routines, which may eventually lower burnout rates^25^.

## Conclusion

This investigation reveals the prevalence of migraine among medical students, which affects their general well-being and academic achievement. Effective management requires early diagnosis and treatment by licensed medical experts^2^. Technology-based therapies, such as apps for tracking migraines and relaxation, have the potential to help students control their condition. Moreover, the need to employ a multifaceted strategy in medical education is underscored. This involves providing access to healthcare resources, encouraging a supportive environment through faculty training and flexible policies, and launching educational programs to manage migraines^19^.

By including teaching about migraines in medical curricula, future doctors will be better prepared to treat patients who face similar difficulties and manage their own migraines. Furthermore, dedication to student success can be seen in supporting rules that put the needs of students first, such as flexible exam scheduling or excused absences for migraines. A comprehensive strategy to treat migraine in medical students can be accomplished by promoting cooperation among medical schools, student organizations, healthcare providers, and governmental organizations. Improved knowledge, easier access to resources, and the creation of research-based solutions to promote students’ well-being and future success as healthcare professionals are all possible outcomes of this partnership^11,19^.

## Ethical approval

Ethics approval was not required for this review.

## Consent

Informed consent was not required for this review.

## Source of funding

None.

## Author contribution

M.S.: conceptualisation; data curation; formal analysis; investigation; methodology; supervision; writing—original draft; writing—review and editing. M.U.F.: methodology; writing—original draft; writing—review and editing. M.N.A.A.: methodology; writing—original draft. F.K.: writing—review and editing. T.D.E.A.: supervision; writing—review and editing. G.M.: writing—review and editing.

## Conflicts of interest disclosure

The author declares no conflicts of interest.

## Research registration unique identifying number (UIN)

None.

## Guarantor

1. Muhammad Saeed; D.G. Khan Medical College, Dera Ghazi Khan, Pakistan; saeedmuhammad0102@gmail.com
2. Tangmi Djabo Eric Adrien; Department of Medicine, Université Technologique Bel Campus, Kinshasa, Democratic Republic of Congo; tangmiadrien@gmail.com

## Data availability statement

The datasets for this study can be availed by the reader through the corresponding author (tangmiadrien@gmail.com).

## Provenance and peer review

None.
